# The antipsychotic medication, risperidone, causes global immunosuppression in healthy mice

**DOI:** 10.1371/journal.pone.0218937

**Published:** 2019-06-26

**Authors:** Meghan May, Megan Beauchemin, Calvin Vary, Deborah Barlow, Karen L. Houseknecht

**Affiliations:** 1 Department of Biomedical Sciences, College of Osteopathic Medicine, University of New England, Biddeford, ME, United States of America; 2 Center for Molecular Medicine, Maine Medical Center Research Institute, Scarborough, ME, United States of America; University of Toronto, CANADA

## Abstract

Atypical antipsychotic medications such as risperidone are widely prescribed for diverse psychiatric indications including schizophrenia, bipolar disorder and depression. These medications have complex pharmacology and are associated with significant endocrine and metabolic side effects. This class of medications also carries FDA black box warnings due to increased risk of death in elderly patients. Clinical reports indicate that patients treated with these medications are more susceptible to infections; however, the underlying mechanisms/pharmacology are unclear. We have previously reported that risperidone and it’s active metabolite distributes to the bone marrow in clinically relevant concentrations in preclinical species, leading us to hypothesize that the hematopoietic system may be impacted by these medications. To test this hypothesis, using proteomic and cytokine array technology, we evaluated the expression of genes involved in inflammatory and immune function following short term (5 days) and longer term (4 weeks) treatment in healthy animals. We report that low-dose risperidone treatment results in global immunosuppression in mice, observed following 5 days of dosing and exacerbated with longer term drug treatment (4 weeks). These data are consistent with increased susceptibility to infection in patients administered these medications and have profound implications for the increasing off-label prescribing to vulnerable patient populations including children and the elderly.

## Introduction

Antipsychotic medications are FDA approved for treatment of psychosis associated with schizophrenia and bipolar disorder as well as severe depression and autism-related irritability. These medications have complex pharmacology, antagonizing myriad G protein coupled receptors (GPCRs) including dopamine D2, serotonin 5HT2, alpha adrenergic, histaminergic and muscarinic receptors. It is thought that antipsychotic efficacy is linked primarily to central antagonism of dopamine D2 and 5HT2c receptors [1–2]. Over the past 10–15 years, off-label prescribing of these medications has been increasing for diverse indications such as attention deficit/hyperactivity disorder (ADHD), insomnia, and agitation associated with dementia, despite lack of evidence for efficacy and black box FDA warnings relating to patient safety [3–5]. Antipsychotics are associated with serious side effects including obesity, dyslipidemia, diabetes, increased risk of fractures and bone loss [6–11].

Although the reasons are not well defined, antipsychotic medications are also associated with increased mortality in vulnerable populations receiving off-label prescriptions including children [12] and older adults [13–14]. Clinical data indicate that schizophrenic patients are more susceptible to infections [15]; the role of medications, *per se*, on the increased susceptibility to infections in this population has not been clearly delineated. Off-label antipsychotic use in elderly patients is associated with a higher incidence of urinary tract infections [16], and a prospective study of Parkinson’s disease patients treated with antipsychotics found a 2-fold greater incidence of infection compared to those who are not [13]. Despite these observations, the role of antipsychotic medications in determining susceptibility to infectious disease is not understood. We have previously reported that the antipsychotic drug, risperidone (RIS) distributes to bone marrow in preclinical models (female c57bl/6 mice) following a single oral dose [17]. This drug distribution coupled with the associations of antipsychotic use with increased incidence of infection in patients led us to hypothesize that the bone marrow compartment may be a key target of RIS action, resulting in altered immune function and increased susceptibility to infection. Here we report, using a pre-clinical model of young adult mice, that RIS and the active metabolite, 9-OH-risperidone (paliperidone; PAL) distribute to the marrow compartment, and that acute (5 days) and chronic (4 week) RIS treatment results in global immunosuppression in healthy animals.

## Methods

### Animal care and use

The preclinical model employed for these studies uses 7–8 week old male C57BL/6J mice, fed standard mouse chow (18% protein rodent chow; Envigo) and treated once daily with vehicle (VEH; 0.1% acetic acid, PO) or a low, clinically relevant dose of risperidone for 5 days (Sigma; 0.75 mg/kg, PO) or 4 weeks (Sigma; 1 mg/kg, PO). This drug dose was chosen based upon preliminary PK studies conducted in our laboratory [17] which reflect plasma drug exposure consistent with what is observed clinically [18]. At the end of each treatment period, mice were sacrificed by CO_2_ asphyxiation (5 day treatment) or avertin (2.5% in PBS) injection (4 week treatment). Plasma (EDTA), heart, liver, bone marrow, spleen, and thymus were collected for analysis. All animal study protocols were approved by the University of New England IACUC committee.

### Drug exposure

Concentrations of RIS and the active metabolite, PAL, in plasma and bone marrow were determined by liquid chromatography-tandem mass spectrometry (LC-MS/MS) analysis as previously reported [17]. Briefly, RIS and PAL were extracted from both plasma and bone marrow via protein precipitation with acetonitrile. Separation was accomplished using a Waters XBridge C18 analytical column (3.0 x 50 mm, 3.5 μm). Mobile phase consisted of 0.1% formic acid in purified water (A) and 0.1% formic acid in acetonitrile (B). The flow rate was 0.4 mL/min, and heated to 60°C. Gradient elution was employed, with initial conditions 95% A and 5% B. Solvent composition was held at the initial conditions for 1.0 minutes, and then was ramped over the following 1.5 minutes to 95% B. Composition was maintained at 95% B for 1 minute. RIS and PAL were detected via an Agilent (Waldbronn, Germany) 6460 triple quadrupole mass spectrometer operated in positive ion MRM mode. The following transitions were monitored: RIS (411.2→191.0) and PAL (427.2→207.0). Significant differences between groups were determined by two-tailed T test using GraphPad Prism.

### Measurement of systemic immune markers

Serum concentrations of forty immune markers (cytokines, chemokines, and soluble Intercellular Adhesion Molecule [ICAM]-1) were measured in mice treated with RIS or VEH for 5 days or 4 weeks using the Proteome Profiler Mouse Cytokine Array (R&D Systems, Minneapolis, MN) according to the manufacturer’s procedures. Arrays were imaged using a FluorChem Q instrument and quantified using ImageQuant v. 8.2. Each duplicate array spot representing a unique immune marker was normalized to each of 3 reference spots per array (N = 3 measurements per spot). Normalized measurements were averaged for each spot, and the duplicate spots for each feature were then averaged to generate a total measure for each immune marker. Unique arrays were run for each animal (N = 6 animals per condition [*i*.*e*., 5 day VEH-treated mice 1 hour post-dosing; 5 day RIS-treated mice 1 hour post dosing; 5 day VEH-treated mice 3 hours post-dosing; 5 day RIS-treated mice 3 hours post dosing; 4 week VEH-treated mice 3 hours post-dosing; 5 day RIS-treated mice 3 hours post dosing]). Significant differences between groups for each immune function marker were determined by two-tailed T test using GraphPad Prism. Responses were categorized as follows: acute onset/transient effects (those present at 5 days plus 1 hour of dosing only), early onset/transient effects (those present at 5 days plus 3 hours of dosing only), early onset/persistent effects (those present at 5 days plus 3 hours of dosing and 4 weeks of dosing), late onset effects (those present at 4 weeks of dosing), or chronic effects (early onset/persistent effects plus late onset effects).

### Histology

Spleen, thymus, and femur were collected during necropsy from each animal following 5 days of RIS or VEH. Tissues were post-fixed in 4% paraformaldehyde overnight. Bones were decalcified using 10% EDTA across 3 weeks. Tissues were embedded in paraffin and 5 mm sections were taken for staining using hematoxylin and eosin (H&E) in order to qualitatively assess changes in pathology. Two sections from each animal were examined via brightfield microscopy at 4x, 10x, and 40x magnifications using a Keyence BZ-X710 inverted widefield digital microscope. Images were collected using BZ-X analyzer software.

### Immunological function pathway analysis

Entries for all measured cytokines were accessed from the Kyoto Encyclopedia of Genes and Genomes (KEGG) Database [19]. Pathways for each measured immune marker involved in immune function (N = 27 pathways) or responses to specific infectious diseases (N = 24 pathways) were pulled and analyzed. Pathways involving measured features that are not linked to immune function or infectious disease responses were not included in the analysis. The number of altered cytokines present at wild-type levels (*i*.*e*., that of VEH-treated mice) were tabulated in VEH and RIS treatment groups at each effect stage (acute onset/transient, early onset/transient, early onset/persistent, and chronic). Chronic exposure values reflect the total cytokines that were altered at both 5 days and 4 weeks. Heat maps reflecting the total altered immune factors for each pathway at each effect stage were generated. Each immune factor present at significantly higher levels in one group was allotted proportional wavelength in their assigned color on a scale from 55 nm to 255 nm (VEH = red; RIS = green) (Immune Function Pathway map λ = 21 nm per altered measure; Infectious Disease Response Pathways λ = 33 nm per altered measure). Multiple factors present at significantly higher levels contribute additively to color intensity. Absence of change between groups in immune mediator levels in an effect category are colored black (λ = 0 nm).

### Proteomic analysis

A proteomic analysis of heart tissue was performed as part of a larger study focused on side effects of antipsychotic drugs. Whole hearts and portions of liver from RIS-treated (N = 5) and VEH-treated (N = 4) male mice were homogenized in HTNG lysis buffer (20 mM HEPES, 150mM NaCl, 1.5mM MgCl_2_, 10% glycerol, 1% Triton‐X 100 1mM EDTA, protease‐inhibitor cocktail (Calbiochem). Protein concentration of the supernatant was determined by BCA (pierce). 40 μg of protein was used from each sample. Tryptic digests of protein samples were performed using the ProteoExtract digestion kit (Calbiochem). Tryptic peptides were then separated on a Ultimate RSLC system 3000 (ThermoFisher/Dionex) nanoscale liquid chromatograph and infused onto a 5600 TripleTOF mass spectrometer (Sciex). Sequential window acquisition of all theoretical spectra (SWATH) was used to profile all proteins in each sample using a data-independent acquisition method. A human-specific ion library comprising 4091 proteins was constructed using ProteinPilot software (Sciex). For identification of peptides, multiple fragment ion chromatograms were retrieved from the spectral library for each peptide of interest. These spectra were compared with the extracted fragment ion traces for the corresponding isolation window to identify the transitions that best identify the target peptide. SWATH analysis was performed using PeakView software, and MarkerView software was utilized for principal component analysis and T-test comparisons [20]. Detailed proteomic analysis methods are available at PeptideAtlas (Identifier: PASS01349). The proteomic data were analyzed in the following pairwise comparisons: heart RIS to heart control. An adjusted p-value was calculated for each protein in each pairwise data set using the FDR method. Two significant protein lists were then made for each pairwise data set using the raw and adjusted p-values with a threshold of 0.05. Proteins associated with immune functions or phenotypes showing altered expression during RIS treatment relative to VEH-treated controls were binned and tabulated for this study.

## Results

### Animal health

The dose of drug selected for these studies results in total plasma drug concentrations that fall in the low end of the clinical range and causes no significant change in feeding, body weight or general behavior as previously published [9, 17, 21]. In all treatment cohorts, animals appeared healthy and gained weight, as expected. In the 4 week treatment cohort, VEH animals (n = 8) weighed 23.3 +/- 0.95 on day 1 and 25.6 +/- 1.16 g at the culmination of the study. RIS animals (n = 9) weighed 23.1 +/- 0.93 g on day 1 and 25.2 +/- 1.91 g at the end of the study. In the 5 day treatment cohort, animals treated with VEH and RIS for 5 days averaged 24.33 +/- 0.57 and 24.1 +/- 0.87 g on d1 and 24.8 +/- 0.45 and 24.02 +/- 0.51 g on d5, respectively.

### Drug distribution to the marrow compartment

**Fig 1:**
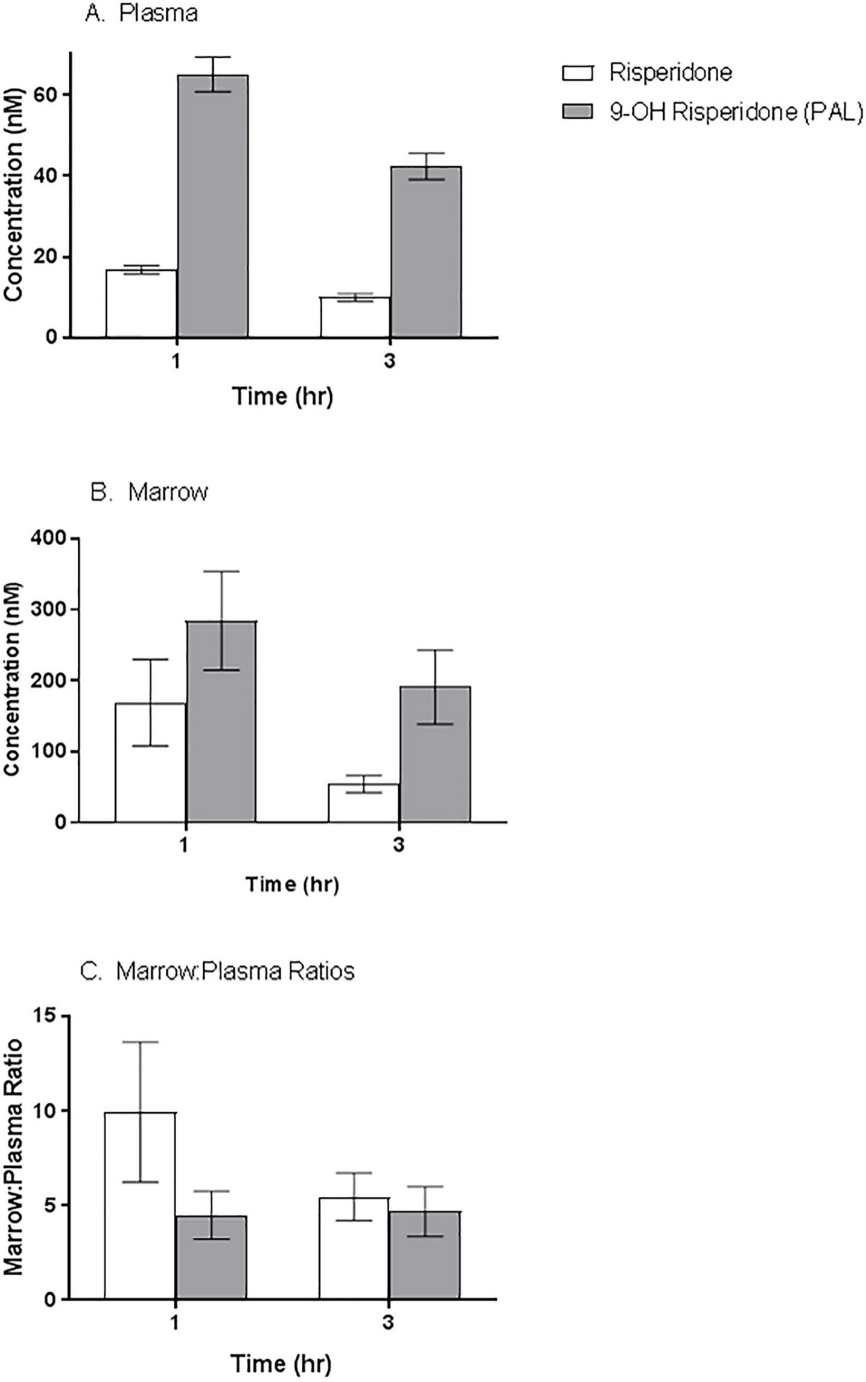
Risperidone/Paliperidone concentrations after 5 days of treatment. Plasma and bone marrow concentrations of risperidone (white bars) and its active metabolite paliperidone (grey bars) were measured 1 and 3 hours post dosing. Paliperidone concentrations expectedly exceeded risperidone in both plasma and bone marrow. Concentrations of risperidone and paliperidone were found in at least 10-fold excess in bone marrow compared to plasma at both time points.

RIS and the active metabolite (PAL) distributed to marrow of male mice following 5 days of dosing, and concentrations were higher in marrow than in plasma at 1 and 3 hours post-dose (10–15 fold higher in marrow vs. plasma; *P*<0.05; (Fig 1). Samples from vehicle treated animals had no drug or metabolite concentrations detectable above the limit of quantification (LOQ) of the assay (1 nM). These data are consistent with our previous report of RIS and PAL distribution to bone marrow of female mice following a single oral dose [17].

### Detection of systemic immune markers

**Fig 2:**
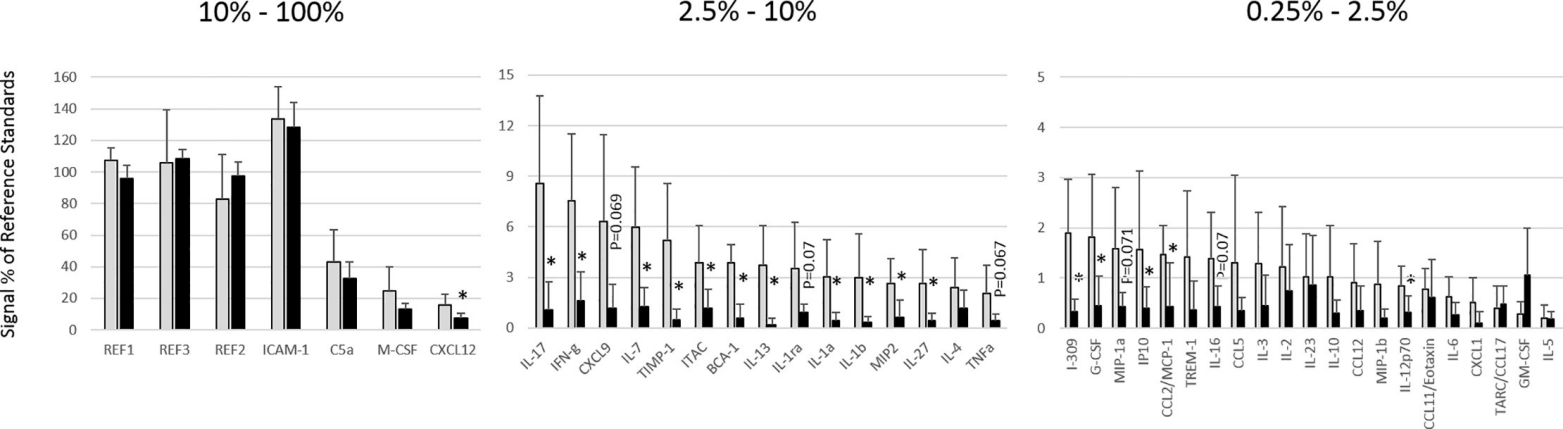
Immunological parameters after 4 weeks of treatment. Levels of 40 different cytokines or chemokines were measured by normalized pixel density (% of reference spots, Y axis) after 4 weeks of continuous risperidone (black bars) or vehicle (grey bars) treatment. Seventeen cytokine/chemokines were significantly (*, *P*<0.05) reduced in risperidone-treated mice relative to vehicle-treated mice. An additional 5 markers were substantially reduced, with *P* values ranging between 0.05 and 0.1 (marked). No markers were significantly elevated in risperidone-treated mice.

**Fig 3:**
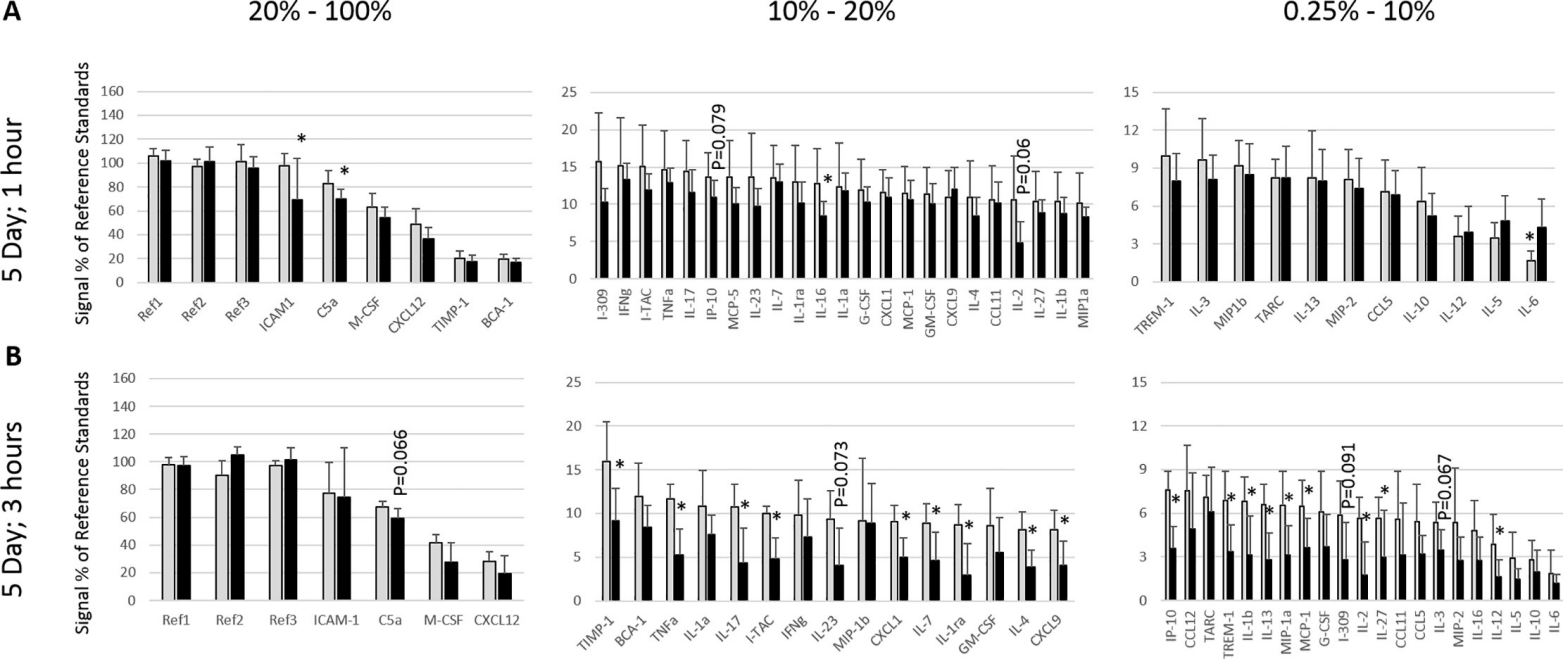
Immunological parameters after 5 days of treatment. Levels of 40 different cytokines or chemokines were measured by normalized pixel density (% of reference spots, Y axis) after 5 days of continuous risperidone (black bars) or vehicle (grey bars) treatment. Measurements were made 1 hour (A) and 3 hours (B) after the fifth dose. Only ICAM1, C5a, and IL-16 were significantly (*, *P*<0.05) 1 hour post treatment; however, 18 cytokine/chemokines were significantly (*, *P*<0.05) reduced by 3 hours. Two or 4 additional markers were substantially reduced with *P* values ranging between 0.05 and 0.1 (marked) at 1 and 3 hours, respectively. IL-6 was significantly elevated in risperidone-treated mice at 1 hour post treatment, but the effect was lost by 3 hours. Markers that were reduced at 1 hour (ICAM1, C5a, and IL-16) all rebounded to normal levels by 3 hours; however, both markers that were trending downward at 1 hour (IP10 and IL-2) were significantly reduced in treated mice by 3 hours.

Plasma concentrations of 40 cytokines or chemokines were measured in plasma from mice treated for 4 weeks or 5 days with RIS or VEH. Significant changes or trends between VEH- and RIS-treated groups were observed for 31 of 40 measured immune markers at at least one effect stage (*i*.*e*., acute onset/transient, early onset/transient, early onset/persistent, or late onset; Table 1; Figs 2 and 3). Twenty-one (>50%) of the evaluated immune markers were diminished relative to VEH controls after only 5 days of RIS treatment, and were persistently diminished through 4 weeks of RIS treatment.

**Table 1:** Cytokine profiles in a preclinical model of RIS therapy.

Cytokine/Chemokine	5 Day Treatment		4 Week Treatment	Temporality of Effect
	1 hour	3 hour		
ICAM-1	↓	↔	↔	Acute, transient
IL-16	↓	↔	↓^a^	Acute, transient
IL-6	↑	↔	↔	Acute, transient
C5a	↓	↓^a^	↔	Acute/Early, transient
IL-2	↓^a^	↓	↔	Acute/Early, transient
IL-3	↔	↓^a^	↔	Early, transient
CXCL1	↔	↓	↔	Early, transient
IL-4	↔	↓	↔	Early, transient
IL-23	↔	↓^a^	↔	Early, transient
TREM-1	↔	↓	↔	Early, transient
MIP-1α	↔	↓	↓^a^	Early onset, chronic
TNF- α	↔	↓	↓^a^	Early onset, chronic
I-309	↓^a^	↓^a^	↓	Early onset, chronic
IL-17	↔	↓	↓	Early onset, chronic
IL-7	↔	↓	↓	Early onset, chronic
CXCL9 (MIG)	↔	↓	↓^a^	Early onset, chronic
IL-1ra	↔	↓	↓^a^	Early onset, chronic
IL-1β	↔	↓	↓	Early onset, chronic
IL-27	↔	↓	↓	Early onset, chronic
TIMP1	↔	↓	↓	Early onset, chronic
ITAC	↔	↓	↓	Early onset, chronic
IL-13	↔	↓	↓	Early onset, chronic
IP10 (CXCL10)	↔	↓	↓	Early onset, chronic
MCP1 (CCL2)	↔	↓	↓	Early onset, chronic
IL-12	↔	↓	↓	Early onset, chronic
CXCL12	↔	↔	↓	Late onset, chronic
INFγ	↔	↔	↓	Late onset, chronic
BCA-1	↔	↔	↓	Late onset, chronic
IL-1α	↔	↔	↓	Late onset, chronic
CXCL2 (MCP2)	↔	↔	↓	Late onset, chronic
G-CSF	↔	↔	↓	Late onset, chronic
M-CSF	↔	↔	↔	Unchanged
GM-CSF	↔	↔	↔	Unchanged
IL-5	↔	↔	↔	Unchanged
CCL5 (RANTES)	↔	↔	↔	Unchanged
IL-10	↔	↔	↔	Unchanged
TARC	↔	↔	↔	Unchanged
MIP-1β	↔	↔	↔	Unchanged
CCL11 (eotaxin)	↔	↔	↔	Unchanged

^a^Feature was trending toward significance in alteration, with a *P* value between 0.1 and 0.05

### Histology

**Fig 4:**
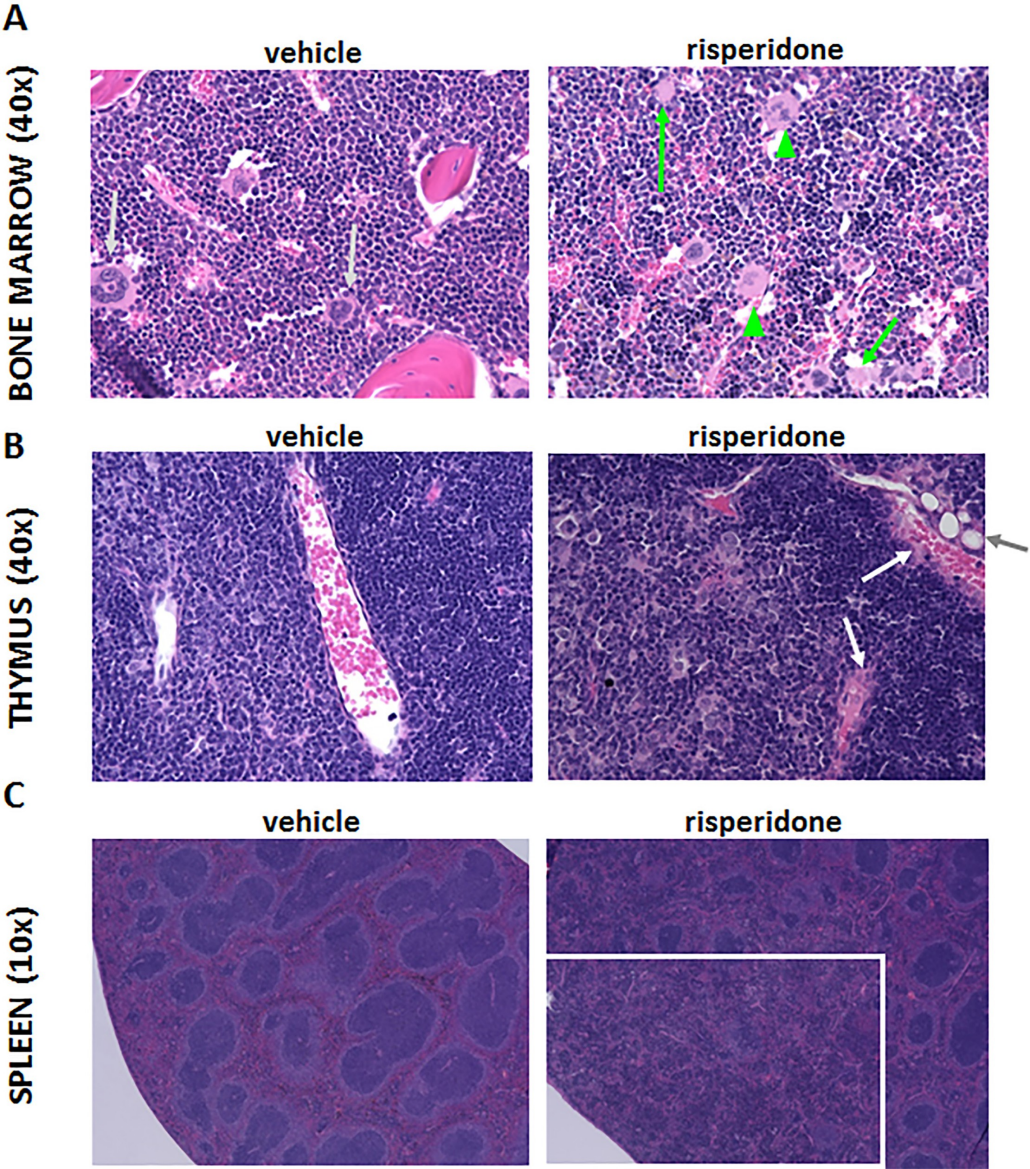
Histopathological changes after 5 days of treatment. Hematoxylin and eosin staining of bone marrow, thymus, and spleen showed changes across cohorts between risperidone- versus vehicle-treated mice. Bone marrow sections (A) from risperidone-treated mice revealed myeloid dysplasia (green arrow heads) and necroptotic cells (green arrows), whereas myeloid progenitors from vehicle-treated mice featured normal myeloid progenitors (silver arrows). Thymus sections (B) from risperidone-treated mice showed hyaline staining, most notably along vessel walls (white arrows), and early steatosis (grey arrow). Cellular changes were not apparent in the spleen between risperidone- and vehicle-treated mice; however, sections viewed at low magnification (C) show a disorganization of white pulp (boxed area) with loss of marginal zones and germinal centers in risperidone-treated mice.

H&E staining of bone marrow, thymus, and spleen showed changes across cohorts between RIS- and VEH-treated mice (Fig 4) after 5 days of dosing. Bone marrow sections from RIS-treated mice did not have evidence of hypocellularity or overt defects in lymphoid cells. However, sections revealed extensive myeloid dysplasia and nectroptotic myeloid lineage cells (Fig 4A). Thymus sections from RIS-treated mice showed hyaline staining throughout the tissue, most notably along vessel walls. Many animals had visible fat infiltration in the thymus, indicating the tissues were in the early stages of steatosis (Fig 4B). There were no apparent changes at the cellular level in the spleen; however, the overall organization of the white pulp was altered in RIS-treated mice, featuring loss of marginal zones and germinal centers (Fig 4C).

### Immune function pathway analysis

**Fig 5:**
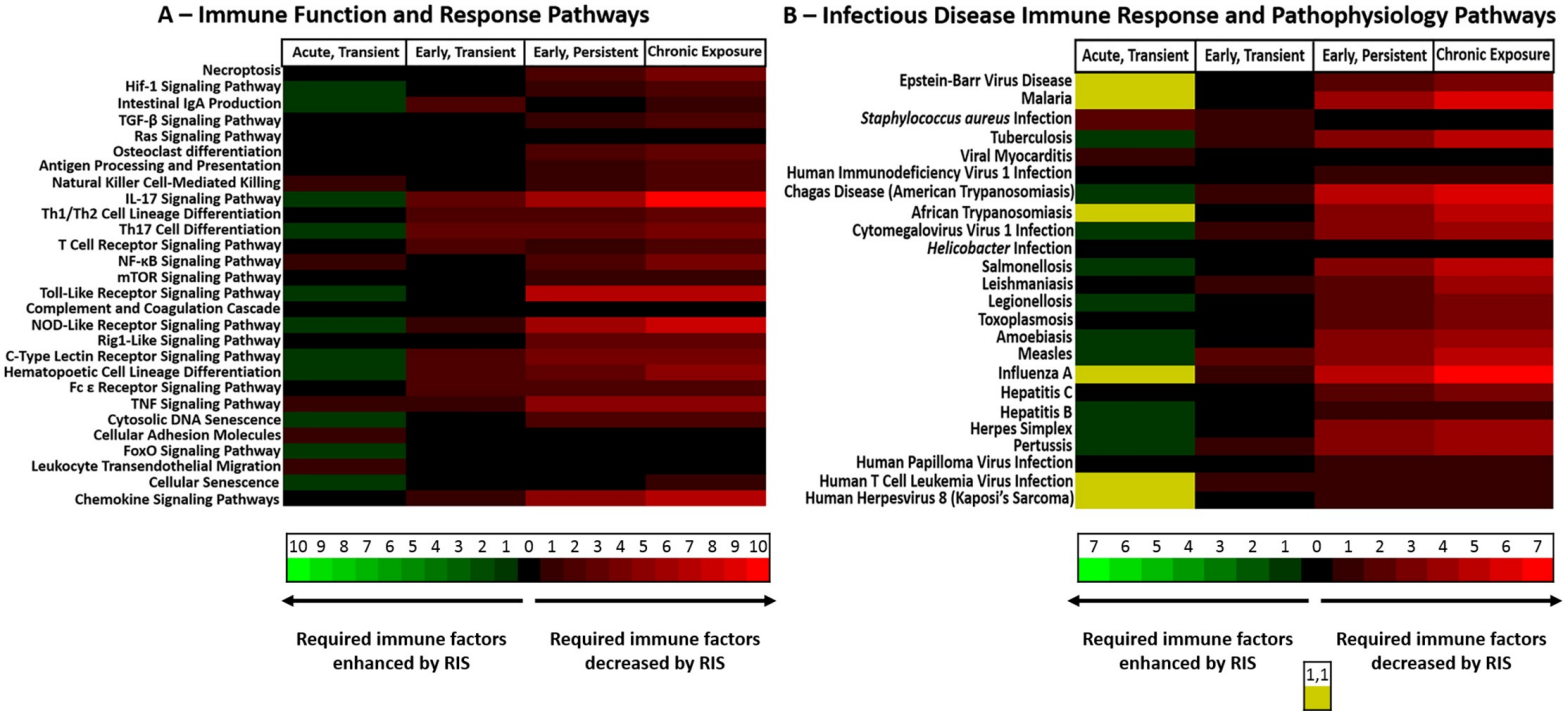
Immune dysregulation and infectious disease susceptibility during risperidone treatment. Cytokines measured in this study are integral to several immune function and infectious disease response pathways in the KEGG database. The number of altered cytokines present at wild-type levels at each effect stage (acute/transient effect, early/transient effect, early/persistent effect, or chronic effect) were tabulated in VEH-treated (red) and RIS-treated (green) mice. Chronic exposure measurements reflect the total cytokines that were altered at both 5 days and 4 weeks. Absence of altered cytokines in an effect category are colored black (λ = 0 nm). The intensity of red reflects the number of immune markers significantly decreased during RIS treatment, and consequently the strength of function in VEH-treated mice relative to function in RIS-treated mice, and vice versa regarding green intensity showing an expected enhancement of pathway function. Twenty-seven immune function pathways (A) included measured cytokines, and alterations were apparent in all but the Ras signaling pathway. A small number of pathways were transiently enhanced or depressed by RIS treatment before recovering fully or subsequently becoming dysregulated. Twenty-one pathways include depressed cytokines after five days of RIS treatment, and nineteen were further dysregulated by four weeks. Twenty-three of the twenty-seven immune function pathways had reduction of at least one cytokine after four weeks of RIS treatment. Twenty-four infectious disease response pathways (B) included measured cytokines, and all but *Helicobacter* infection involve altered cytokines. RIS-treatment would increase susceptibility to *Staphylococcus aureus* and viral myocarditis acutely after dosing, but would not confer increased risk by continuous therapy. RIS could transiently enhance responses to ten infections, though any enhancement would be offset by parallel reductions of a different immune mediator during responses in six others (shown as equivalent red and green wavelengths, appearing as yellow). After five days of treatment, RIS treatment causes dysregulation of pathways involved in defense against twenty-one different infectious diseases. All disruptions persisted through 4 weeks of treatment, and sixteen of the twenty-one were further dysregulated followed longer drug treatment.

Cytokines measured in this study are integral to several immune function and infectious disease response pathways in the KEGG database, and the complete panel of cytokines with serum levels altered during RIS treatment are shown via heat maps (Fig 5). The intensity of red is directly proportional to the number of measured immune mediators depressed in RIS-treated mice relative to VEH-treated mice for each pathway. The intensity of green is directly proportional to the number of mediators that are increased in RIS-treated mice for each pathway. Alterations in at least one measured immune marker level were induced by RIS treatment in all examined pathways with the exception of the Ras signaling pathway. A small number of pathways were transiently enhanced or depressed by RIS treatment before recovering fully or subsequently becoming dysregulated. Twenty-one pathways include depressed immune markers after five days of RIS treatment, and nineteen were further dysregulated by four weeks. Twenty-three of the twenty-seven immune function pathways had reduction of at least one cytokine after four weeks of RIS treatment (Fig 5A). Twenty-four infectious disease response pathways included immune markers measured in this study, and all but *Helicobacter* infection involve at least one immune marker altered by RIS treatment. After five days of treatment, RIS treatment caused dysregulation of pathways involved in defense against twenty-one different infectious diseases. All disruptions persisted through 4 weeks of treatment, and sixteen of the twenty-one were further dysregulated followed longer drug treatment (Fig 5B).

### Immunoproteomic changes in the heart and liver during RIS treatment

Twenty-three proteins found in pathways associated with immune functions or infectious disease phenotypes showed altered expression (*P* < 0.05) in the heart during RIS treatment relative to VEH-treated controls after 4 weeks (Table A in S1 Tables). Similarly, twenty-eight proteins found in these pathways showed altered expression in the liver (*P* < 0.05; Table B in S1 Tables). Quantitative raw data have been deposited and can be accessed via PeptideAtlas (Identifier: PASS01349).

## Discussion

The highly prescribed antipsychotic, risperidone, distributes to bone marrow in mice and is associated with global immunosuppression. Initial findings made in the context of a larger study on the side effects of antipsychotic medications showed proteomic changes in the heart and liver involving proteins involved in immune function during RIS treatment. While not primary immunological organs, these changes in the heart and liver indicated that genes encoding proteins that can impact immune responses are directly or indirectly responsive to the presence of RIS (Tables A and B in S1 Tables). In order to validate these findings in a biologically relevant manner, we assessed whether systemic changes in functional immune mediators occur during RIS treatment. Reduced plasma concentrations of multiple cytokines and immune modulators were observed following short term (5 days) and chronic (4 week) dosing. These data are consistent with clinically observed increased susceptibility to infection, and have profound implications for off-label prescribing.

To our knowledge, the current study represents the most comprehensive analysis of antipsychotic-induced effects on immune system mediators *in vivo*, by evaluating concentrations of forty distinct markers at multiple time points following RIS treatment (Figs 2 and 3). Plasma concentrations of cytokines beyond normal limits have been reported several times in drug-naive schizophrenic patients, leading to the hypothesis that the disease has a large inflammatory component that is as yet poorly defined [22–25]. This is further supported by newer reports of the responsiveness of schizophrenia to anti-inflammatory drugs [26–28]. Apparent normalization of some cytokine levels following treatment of schizophrenia with antipsychotic drugs as well as *in vitro* demonstrations of their ability to impact pro- and anti-inflammatory cytokines [29–32] has led to speculation and analysis that immunomodulation is a previously unrecognized component of their therapeutic effect for patients experiencing psychosis [33]. The notion of therapeutically relevant immunomodulation has been applied to lethal inflammatory conditions such as bacterial sepsis, wherein the typical antipsychotic drug trifluoperazine showed an improved survival and reduced organ damage in a preclinical model [34]. This reciprocal potential for drug repurposing is reflective of the bi-directional interactions between the immune and nervous systems, notable here for the demonstrated involvement of dopaminergic and serotonergic signaling in both processes. Previous studies evaluating the immunomodulatory effects of typical and atypical antipsychotic drugs have often been limited in scope to the evaluation of single or small numbers of cytokines. Most explorations of cytokine levels in humans have focused on schizophrenic patients, where mechanistic evaluations of the impact of antipsychotic drugs on previously well-regulated immune functions are strongly confounded due to the inherently dysregulated inflammatory responses in these patients. A small number of studies evaluating the impact of cytokine release by immune cells such as dendritic cells and peripheral blood mononuclear cells have been carried out *in vitro* [29, 31]. In some instances these studies led to conflicting findings with data from human patients; however, the inherent limitations of immune signal measurement *in vitro* and the complex inflammatory profile of schizophrenic patients make these discrepancies predictable.

Our previous studies showing that RIS and the active metabolite, PAL, distribute to bone marrow raised the distinct possibility of direct action on the hematopoietic system via GPCR antagonism [17]. This necessitated a prospective, *in vivo* study where confounding variables such as underlying inflammatory disorders or concurrent infections could be tightly controlled. Concentrations of RIS and PAL in the marrow compartment are higher than observed in plasma and exceed the Kd for binding to GPCRs known to be targeted by these drugs, including dopaminergic and serotinergic receptors. Changes in inflammatory responses can be driven by changes in dopaminergic and serotonergic signalling through various mechanisms [35–43], and therefore antagonism of these receptors makes some level of inflammatory modulation predictable. We have previously reported [17] that dopamine is present in the marrow compartment and that RIS exhibits direct effects on osteoclast differentiation, demonstrating that bone marrow is a pharmacologically relevant compartment for RIS action. Given the expression of some of the target receptors within the hematopoietic system and the interaction between dopaminergic and serotonergic signalling and inflammation, pharmacologically active drug concentrations in bone marrow are likely significant. Histopathologic changes were apparent in bone marrow after only 5 days of drug exposure, supporting the notion that RIS is acting on cells that regulate myeloid cell maturation either directly or indirectly. While neutropenia is a known side effect of clozapine [44–46], it is increasingly seen as a complication of other atypical antipsychotic drugs. A prospective study and several case reports describe either neutropenia or panleukopenia induced by RIS treatment of patients treated either for psychosis or off-label uses [47–51]. Our findings of myeloid dysplasia and necroptosis in bone marrow (Fig 4A) following RIS exposure are consistent with these clinical findings, and provide a mechanism for the observed effect in patients.

Histopathologic changes observed within the thymus (Fig 4B) and spleen (Fig 4C) after 5 days of RIS treatment are indicative of systemic impacts on distal lymphoid organs. Changes seen within the thymus (*i*.*e*., steatosis, hyaline staining) are consistent with those seen in natural thymic involution and atrophy due to aging [52]; however, these changes were accelerated in RIS-treated animals as indicated by their absence in VEH-treated mice. Clinical outcomes of thymic atrophy include immune dysregulation and lowered immunological function [53]. Immune function pathways related to T cell maturation/differentiation were among the most heavily impacted by RIS treatment (Fig 5A, Chronic Effects), strongly suggesting that dysregulated cytokine signalling during RIS treatment is a mechanism for the observed thymic pathology.

The effects observed in the current study are seen in a pre-clinical model using unchallenged animals without a confounding disorder that impacts immune signalling. Impacts on immune function by RIS treatment seen here are predictive of impacts on non-schizophrenic patients who are prescribed RIS off-label. Interactions between immune function pathways and those describing the pathophysiology of chronic diseases such as type II diabetes mellitus and nonalcoholic fatty liver disease are likely to be similarly impacted by the observed reduction of certain inflammatory mediators (*e*.*g*., TNF-α) in RIS-treated mice. It is notable that many of the clinical features of these diseases such as insulin resistance and dyslipidemia are known side effects of long-term antipsychotic use [6, 7, 10]. The immune markers measured are part of the mapped immune responses to twenty-four different infectious diseases (Fig 5B), and twenty-one of these pathways were chronically disrupted with multiple diminished cytokines after only 5 days of RIS treatment. It is important to note that the KEGG database has mapped responses to a total of twenty-seven infectious diseases, and therefore RIS treatment dysregulates 85% (78% chronically; 7% transiently) of the mapped infection defense responses. Disrupted response pathways during pertussis, measles, influenza A, and hepatitis B would be particularly acute in children and adolescents [54–57], who as a population can experience off-label use of antipsychotics in the treatment of ADHD. Disruption in the responses to legionellosis, influenza A, and *Staphylococcus aureus* infection, all of which are significant causes of morbidity and mortality in nursing home residents [58–60], are also predictable. Off-label antipsychotic drug use in nursing homes has been reported to be as high as 37% of patients (with and without dementia) [61]. Taken together, this suggests that 1 in 3 nursing home residents would be at elevated risk for infections specifically known to cause adverse outcomes in their population.

Based on our preclinical model, it is predictable that reductions in cytokine levels during RIS treatment would disrupt responses to the overwhelming majority of infectious diseases in immunocompetent individuals, and could further compound function in immunocompromised individuals. This is consistent with clinical observations of elevated levels of infection in both schizophrenic and non-schizophrenic patients treated with antipsychotic drugs, and the global immune dysregulation induced by RIS suggests a mechanism that can account for this phenomenon. Consideration of off-label prescribing in children with ADHD, a broadly immunocompetent population, and older adults with insomnia or dementia, a broadly immunocompromised population, should therefore be weighed strongly against the lack of evidence for efficacy of antipsychotics for these conditions.

## Supporting information

S1 Tables Proteomic Changes in Heart (Table A) and Liver (Table B) Following RIS Treatment (4 Weeks).
(DOCX)
