# Supplementary material for: The antipsychotic medication, risperidone, causes global immunosuppression in healthy mice
Source: PLoS One. 2019 Jun 26;14(6):e0218937. doi: 10.1371/journal.pone.0218937 (PMC6594635; doi:10.1371/journal.pone.0218937)
Supplement: S1 Tables — Proteomic Changes in Heart (Table A) and Liver (Table B) Following RIS Treatment (4 Weeks). (DOCX) [file pone.0218937.s001.docx]

**S1 Table A. Proteomic Changes in Heart Following RIS Treatment (4 Weeks)**

| **Protein** | **Log2 Ratio (RIS:VEH)** | ***P* Value^a^** | **Associated Phenotype Pathways (Immune Functions)** |
| --- | --- | --- | --- |
| GC | 0.039 | 0.0000172 | Inflammation |
| SRA1 | -0.104 | 0.00137 | Phagocytosis by antigen-presenting cells |
| PPIA | 0.108 | 0.00181 | Inflammation; HIV infection; reactivation of latent viruses |
| ZEB2 | 0.339 | 0.00181 | Splenomegaly; T cell counts; natural killer cell counts; dendritic cell counts; myeloid progenitor cell counts; cell cycle regulation (mast cells); latent virus reactivation; human Herpesvirus 4 replication |
| NOTCH2 | -0.082 | 0.00408 | Activation of γδ T cells; Th9 cell maturation; total T cell counts; natural killer cell counts; myeloid cell differentiation; granulopoiesis |
| SMARCC1 | -0.236 | 0.00512 | B cell differentiation |
| PLAUR | 0.218 | 0.00557 | Inflammation; phagocytosis by antigen-presenting cells; leukocyte chemotaxis and migration |
| GJA1 | -0.034 | 0.0066 | Inflammation; Leukopenia; regeneration of lymphoid cells and myeloid cells; dendritic cell communication; natural killer cell communication; hematopoietic progenitor cell trafficking; wound healing; bone marrow atrophy |
| MYH7 | 0.066 | 0.00853 | B cell proliferation |
| NQO1 | 0.023 | 0.011 | Neutropenia; Invasion of megakaryocytes and/or granulocytes; bone marrow hypoplasia; production of Th17 cells; splenomegaly |
| LMNA | 0.028 | 0.011 | Release of human herpesvirus 4 |
| TFRC | 0.066 | 0.0156 | Lymphocyte cell cycle regulation; T cell proliferation; Machupo virus and MMT virus pathogenesis |
| MAP1S | 0.13 | 0.0241 | phagocytosis by antigen-presenting cells; *Salmonella enterica* subsp. *enterica* serovar Typhimurium invasion; Infectivity of HIV |
| DOCK2 | -0.055 | 0.0324 | phagocytosis by antigen-presenting cells; activation of αβT cells; leukocyte chemotaxis/migration |
| GAPDH | 0.063 | 0.0364 | Inflammation; acute respiratory distress syndrome; leukocyte cell death (caspase-independent) |
| DPYD | -0.043 | 0.0425 | Leukopenia; neutropenia |
| CAST | 0.087 | 0.0426 | Inflammation |
| CYP51A1 | -0.064 | 0.0432 | Leukopenia; Toll-like receptor signaling; inflammation; fungal infection (*Candida*; *Tinea*; *Aspergillus*; *Coccidioides*; *Cryptococcus*; *Histoplasma*) |
| SERPINC1 | 0.046 | 0.044 | Acute respiratory distress syndrome; inhibition of *Escherichia coli* and *Pseudomonas aeruginosa*; pathogenesis of dengue hemorrhagic fever |
| ITGA7 | 0.058 | 0.045 | Adhesion of mucosal mast cells |
| MFGE8 | 0.072 | 0.0479 | Neutrophil migration; NET production; inflammation; phagocytosis by antigen-presenting cells; vascular permeability; steatosis; autoantibody production |
| CD14 | 0.142 | 0.0498 | Inflammation; sequestration, release, and shuttling of lipopolysaccharide; peptidoglycan binding; bacterial clearance; monocyte interactions; leukopenia |
| CSNK1A1 | -0.131 | 0.0499 | T cell proliferation |

^a^*P* values were derived by two-tailed T test between values from RIS-treated mice and VEH-treated mice

**S1 Table B. Proteomic Changes in Liver Following RIS Treatment (4 Weeks)**

| **Protein** | **Log2 Ratio (RIS:VEH)** | ***P* Value^a^** | **Associated Phenotype Pathways (Immune Functions)** |
| --- | --- | --- | --- |
| MYH9 | 0.132 | 0.0000433 | Eosinophilia; agranulocyte adhesion/diapedesis; lysosome exocytosis; thrombocytopenia |
| ELAVL1 | -0.05 | 0.00927 | Prostaglandin synthesis; leukocytosis |
| RACK1 | -0.046 | 0.0121 | CCR3 signaling (eosinophils); CCR5 signaling (macrophage) |
| INPP5D | 0.086 | 0.0131 | Phagosome formation/fusion; NK, mast cell degranulation/proliferation; B cell anergy; T cell responses |
| EPHA2 | 0.145 | 0.0177 | Leukocytosis |
| TPT1 | -0.039 | 0.0201 | Eosinophil recruitment |
| PDGFRB | 0.089 | 0.0203 | Eosinophilia; leukocytosis; NK, mast cell degranulation/proliferation |
| ITGA3 | 0.057 | 0.0205 | Granulocyte, agranulocyte adhesion/diapedesis; phagosome formation/fusion; leukocyte extravasation signaling; prostaglandin synthesis |
| GNAS | 0.024 | 0.0219 | CCR3 signaling (eosinophils); CCR5 signaling (macrophage); T cell responses |
| LAMP1 | 0.068 | 0.0242 | Cancer cell killing; lysosome exocytosis; T cell responses |
| CTSS | 0.029 | 0.0292 | Eosinophilia; B cell responses; antigen presentation (B cells) |
| AFDN | 0.055 | 0.0305 | Leukocyte extravasation signaling |
| ADIPOQ | 0.126 | 0.0309 | Eosinophilia; NK, mast cell degranulation/proliferation; prostaglandin synthesis; myeloid cell aggregation; hematopoietic cell differentiation |
| HSD3B7 | -0.037 | 0.0311 | B cell responses |
| PRKCA | 0.182 | 0.0314 | Phagosome formation/fusion; agranulocyte adhesion/diapedesis; leukocyte extravasation signaling; CCR3 signaling (eosinophils); CCR5 signaling (macrophage); cancer cell killing; prostaglandin synthesis; leukocytosis; hematopoietic cell differentiation |
| MMP12 | 0.075 | 0.0333 | Granulocyte, agranulocyte adhesion/diapedesis; leukocyte extravasation signaling; eosinophilia |
| ANXA2 | -0.042 | 0.0348 | Infectivity of Enterovirus 71 |
| HSPB1 | 0.06 | 0.0352 | Granulocyte adhesion/diapedesis |
| SEPT11 | 0.156 | 0.0374 | Infectivity of *Listeria monocytogenes* |
| IL1RAP | 0.075 | 0.0398 | Eosinophilia; agranulocyte, agranulocyte adhesion/diapedesis; T cell responses |
| KLKB1 | 0.067 | 0.0412 | Myeloid cell aggregation; wound healing |
| IGHG1 | 0.062 | 0.0419 | Phagosome formation/fusion; eosinophilia; cancer cell killing; eosinophil recruitment; Inhibition of *Bacillus anthracis*; leukocyte cytolysis; inflammation |
| DGKZ | 0.128 | 0.0427 | NK, mast cell degranulation/proliferation; cancer cell killing; T cell responses |
| MSN | -0.082 | 0.0434 | Granulocyte, agranulocyte adhesion/diapedesis; leukocyte extravasation signaling |
| MYL4 | 0.069 | 0.0439 | Agranulocyte adhesion/diapedesis |
| HPGD | 0.023 | 0.0446 | Prostaglandin metabolism/turnover |
| SMARCC1 | 0.099 | 0.0468 | B cell differentiation |
| ROCK2 | 0.128 | 0.0488 | CCR3 signaling (eosinophils); leukocyte extravasation signaling |

^a^*P* values were derived by two-tailed T test between values from RIS-treated mice and VEH-treated mice
